# Have the Pilot County Hospitals’ Service Capability Been Improved Since the Healthcare Reform? An Analysis of 370 Hospitals in China

**Published:** 2019-03

**Authors:** Yinhong Dong, Xingyi YANG, Pengqian FANG, Zhengqiong PAN, Zhenni LUO

**Affiliations:** 1. School of Management, South-Central University for Nationalities, Wuhan, China; 2. School of Medicine and Health Management, Tongji Medical College, Huazhong University of Science & Technology, Wuhan, China; 3. School of Health Management, Guangzhou Medical University, Guangzhou, China

**Keywords:** County hospitals, Service capability, Evaluation, Healthcare reform, China

## Abstract

**Background:**

County public hospital reform is one of the major tasks proposed in Chinese Healthcare Reform., and the evaluation of hospital reform effectiveness is very important and beneficial since it helps the government to understand the current situation of pilot county public hospitals and smoothly start the reform in all county hospitals.

**Methods:**

This study used hospitals data from 2009 to 2012 to evaluate the effectiveness of county public hospital reform through comprehensive service capability. Descriptive analysis method was used, and factor analysis method was used to extract the main factors associated with service capabilities as well as to calculate a composite score. The t-test of two independent-samples methods was used to comparison analyze.

**Results:**

The differences of common factor scores (hospital scale and service capacity, treatment quality, service quality, and services efficiency) between pilot and non-pilot hospitals were not statistically significant (*P*>0.05). The service capability score in 2012 was better than that in 2009 either in pilot or non-pilot group (*P*<0.05). The pilot hospitals’ service capability score was better than that in non-pilot groups either in 2010 or 2012 (*P*<0.05). However, the differences from 2009 to 2012 of service capability score between pilot and non-pilot hospitals were not statistically significant.

**Conclusion:**

The comprehensive service capability of both pilot and non-pilot group all got improvement. However, county public hospital reform did not significantly play a due good role in improving the service capability in pilot group. The reform was helpful to improve the hospital current situation, but it has not completely achieved policy objectives in the sample hospitals of this study.

## Introduction

China has the largest population in the world, and over 50% of its population lives in rural areas. Therefore, the Chinese government must improve the health status of its rural population (1). A three-tiered medical system (county-town-village) was established in the rural areas of China. At the village level, which is the bottom of the three-tiered medical system, rural doctors (“barefoot doctors”) provide basic curative and preventive medical services for villagers in their own clinics. County hospitals are also responsible for providing training and guidance to township health centers, in addition to the supervision of countywide health services (2). In 2012, China had 10940 county hospitals, 37000 township health centers, and 653000 village clinics. Every county has at least one county hospital (3) and almost the entire rural population has access to essential health services at a reasonable cost through the three-tiered medical system.

Before 2009, dwindling financial support from the government caused many rural health facilities to suffer from financial hardships, causing them to spend very little on the purchase of medical equipment and training for health workers (4). Certain market elements forced many of the better-trained health workers to move from rural facilities to urban areas (5), leading to a decline in the service capability of rural health facilities. Increasing distrust and chronic underutilization of rural health clinics occurred. Restricted access to urban medical services further highlighted social issues in the country. Using the occupancy rate as an example, the rate in urban hospitals averaged 85%–90% but averaged less than 50% in county hospitals (1, 6). The differences in resource allocation in rural and urban areas increased. Moreover, while the number of beds and health professionals increased remarkably in urban hospitals, these numbers declined noticeably in rural hospitals over the same period (1). As inequity and the huge health gap became increasingly significant, the Chinese government had to improve its health sector.

In Mar 2009, the Chinese government has launched formally Healthcare Reform with an aim of establishing a more equitable health system (7) and a focus on gradually achieving the goal, for example, everyone has access to basic medical care and health services, as well as improving medical and health services at the grass-roots level. County hospitals are essential to the reform as the main provider of healthcare for rural population and as the top tier in the three-tiered medical system (8).

Hundreds of county-level public hospitals were selected to participate in a pilot project by Chinese National Ministry of Health and government. Some of the main measures of this pilot project included clearly stated roles and functions of county public hospitals, increased government investment, and the establishment of coordinated, unified and effective administrative mechanisms. Governments at all levels increased health investment of each county to improve infrastructure, health workforce, medical equipment, discipline construction, salary and reward of medical workers, basic pharmaceuticals policy, basic medical insurance and so on.

In China, the effectiveness of the reform evaluated from different perspectives. Some studies evaluated the effectiveness through operational efficiency and productivity of county hospital and showed those have gone down (9–11). The effectiveness through patient’s medical expenses and cost of medicine evaluated and those have fallen to some extent as health reform starts (12,13). Some studies evaluated the effectiveness through patients’ satisfaction (14,15) and medical staffs’ comments about the reform (16,17). Patients’ satisfaction was not throughout improved with the implementation of the reform, and some medical staffs hold negative comments about the reform effectiveness in pilot county hospitals. Some studies evaluated the effectiveness through other various variables, such as the number of beds, service infrastructure development, work-force development, medical service quality and efficiency (18–26).

However, there was almost no effectiveness evaluation through comprehensive service capability of public county hospitals before and after the reform. Enhancing the service capability of public county hospitals was one of the key goals pursued by policy makers to ensure that the majority of the rural population would be guaranteed access to basic health services and have common illnesses be treated locally at the rural healthcare level. Therefore, building a comprehensive service capability evaluation index system would assist county hospital reform and development.

This study aimed to find the conditions of service capability, evaluate the improvement of service capability of pilot county hospitals after the healthcare reform in China. It also can help the government to understand the current situation of pilot county public hospitals and smoothly start the reform in all public hospitals.

## Methods

The study did not involve human participants, specimens or tissue samples, or vertebrate animals, embryos or tissues. The Ministry of Health approved this retrospective study and granted permission to the analysis of data.

### Sampling

By considering both economic and social conditions, 18 representative provinces or cities were chosen across the country for county hospital reform by Chinese Ministry of Health, with 185 pilot county hospitals from 18 provinces or cities selected as an experimental group by county hospital reform policy and 185 non-pilot hospitals selected as a control group by research group. Selection criteria for the control group are as follows: we chose one pilot hospital as a reference and also considered the geographic proximity, similar size, and per capita GDP closest to the specific pilot hospital in 2009. This study has a total sampling of 370 county-level hospitals.

We collected data from the Chinese Ministry of Health for the 370 county hospitals from 2009 to 2012 and included indicators, such as hospital bed size, number of medical staff, workload of hospital departments, hospital efficiency, and quality of medical care.

### Methods of data analysis

Through literature analysis (18–26) and expert consultation, and after considering the availability of data, we screened the related indicators with county hospital service capabilities. The descriptive analysis method was used; factor analysis method was used to extract the main factors associated with service capabilities of county hospitals and to calculate a composite score of the serviceability of county hospitals. The *t-*test of two independent-samples methods was used to comparison analyze.

### Service capability indicators of county hospitals

Eleven indicators related to the service capabilities of county hospitals were selected through literature analysis and expert consultation. These indicators include the actual number of open beds (X1), number of medical staff (X2), number of outpatient and emergency patients per year (X3), number of operations per year (X4), number of discharged patients per year (X5), cure rate (X6), improvement rate (X7), clinical and pathological diagnostic accuracy rate (X8), outpatient and hospital admission diagnostic accuracy rate (X9), bed turnover rate (X10), and average length of stay (X11). All statistical indicators are explained below:

1. Actual number of open beds (X1): the total number of sickbeds opened in a hospital.
2. Number of medical staff (X2): the total number of medical staff in a hospital
3. Number of outpatient and emergency patients (X3): the number of non-hospitalized patients is a summation of the number of outpatient and emergency patients.
4. Number of operations per year (X4): the total number of operations performed in a hospital in one year.
5. Number of discharged patients (X5): all patients discharged from the hospital, including all people listed in the “cured,” “improvement,” and “not cured” groups after treatment.
6. Cure rate: the frequency of patients who become healed after treatment (X6). Cure rate= (number of cured cases/number of discharged patients) × 100%.
7. Improvement rate(X7)= (number of improved cases/number of discharged patients) × 100%.
8. Clinical and pathological diagnostic accuracy rate(X8): (number of clinical and pathological diagnosis accuracy/cases of pathological diagnosis) × 100%.
9. Outpatient and hospital admission diagnostic accuracy rate(X9): (number of outpatient and in-patient diagnosis/number of inpatients) × 100%.
10. Bed turnover rate(X10): the number of discharged patients for each bed in a period. According to the National Health Statistical Investigation System (2007), the formula for bed turnover rate is the number of discharged patients / average number of open beds.
11. Average Length of Stay (ALOS) (X11): the average length of hospital stay for each discharged patient is a comprehensive indicator used in the evaluation of benefit and efficiency, as well as healthcare quality and technological level. “Average length of stay=Total bed days occupied by discharged patients/number of discharged patients.”

### Process of factor analysis

We included indices X1 to X11 in 2009 and 2012 of the 370 county hospitals into the factor analysis.

### KMO and Bartlett’s Test

The KMO test coefficient =0.801>0.5. The significant probability *P*-value of chi-square statistic of Bartlett test is <0.001, the data are highly suitable for factor analysis (Table 1).

**Table 1:** KMO and Bartlett Test

***Kaiser–Meyer–Olk in Measure of Sampling Adequacy***		***0.801***
Bartlett’s Test of Sphericity	Approx. Chi-Square	15044.876
	Df	55
	Sig.	0.000

### Extracting characteristic roots

With characteristic roots >1 as a standard, four characteristic roots were extracted, the cumulative contribution rate of the former four main factors was 73.442%, indicating that the amount of original data has been extracted by 73.442%. Moreover, the original data can be reflected more objectively by the four main factors (Table 2).

**Table 2:** Total Variance Explained

***Component***	***Initial eigenvalues***			***Extracted square and loaded***			***Rotating square and loaded***		
	***Total***	***% of Variance***	***Accumulative %***	***Total***	***% of Variance***	***Accumulative%***	***Total***	***% of Variance***	***Accumulative %***
1	4.221	38.369	38.369	4.221	38.369	38.369	3.647	33.152	33.152
2	1.575	14.320	52.689	1.575	14.320	52.689	1.955	17.770	50.922
3	1.257	11.429	64.118	1.257	11.429	64.118	1.404	12.761	63.684
4	1.026	9.324	73.442	1.026	9.324	73.442	1.073	9.758	73.442
5	.918	8.349	81.791						
6	.623	5.664	87.455						
7	.559	5.081	92.536						
8	.327	2.972	95.508						
9	.242	2.200	97.708						
10	.175	1.590	99.297						
11	.077	.703	100.000						

Extraction Method: Principal Component Analysis

### Extracting the common factor

The typical representative variables of the factor loading matrix were not very prominent and thus were difficult to explain well. A rotated factor model can be built after maximum variance orthogonal rotation. This model shows that the first main factor F1 had higher loads on variables X1 (actual number of open beds), X2 (number of medical staff), X3 (number of outpatient and emergency patients per year), X4 (the number of operations per year), and X5 (number of discharged patients), which were 0.887, 0.883, 0.842, 0.811, and 0.744, respectively. The results showed that F1 reflected the situations of X1, X2, X3, X4, and X5; F1 was labeled the hospital scale and service quantity factor. The second main factor F2 (treatment quality factor) had higher loads on variables X6 (cure rate) and X7 (improvement rate); the former was −0.957 and the latter was 0.953. F2 reflected the situations of X6 and X7. The third main factor, F3, had higher loads on variables X8 (clinical and pathological diagnostic accuracy rate) and X9 (outpatient and hospital admission diagnostic accuracy rate), which were 0.826 and 0.824, respectively.

F3 was called the diagnostic quality of hospital service quality factor. F3 reflected the situations of X8 and X9. The fourth main factor, F4, had higher loads on variables X10 (bed turnover times) and X11 (average length of stay) at 0.894 and −0.449, respectively. F4 reflected the situations of X10 and X11; F4 was called hospital services efficiency factor (Table 3).

**Table 3:** rotated component matrix

***Variable***	***Component***			
	1	2	3	4
X1 number of open beds	.887	.153	.057	−.106
X2 number of medical staff	.883	.185	.062	−.099
X3 number of outpatient and emergency patients per year	.842	.187	.085	−.142
X4 number of operations per year	.811	.073	.071	−.097
X5 number of discharged patients	.744	.053	.015	.133
X6 cure rate	−.187	−.957	−.076	.046
X7 improvement rate	.193	.953	.086	−.035
X8 clinical and pathological diagnostic accuracy rate	.025	.111	.826	−.016
X9 outpatient and hospital admission diagnostic accuracy rate	.110	.021	.824	−.024
X10 bed turnover rate	.078	.043	.046	.894
X11 average length of stay	.263	.121	.092	−.449
Extraction method: principal component analysis.				
Rotation method: varimax with Kaiser normalization.				
A. Rotation to converge in four iterations.				

### Build factor score function, and to calculate factor scores

The coefficient matrix of factor score is shown in Table 4. The common factor is expressed as a linear combination of the original variables. A linear regression method was used to obtain the least squares coefficient estimates, namely the factor score coefficients. We calculate factor scores with factor score coefficients by using the following formula (Table 4).

**Table 4:** Coefficient matrix of component scores

***Variable***	***Components***			
	***1***	***2***	***3***	***4***
X1 Number of open beds	.256	−.045	−.026	−.024
X2 Number of medical staff	.250	−.025	−.025	−.016
X3 Number of outpatient and emergency patients per year	.231	−.022	−.006	−.061
X4 Number of operations per year	.242	−.083	−.003	−.024
X5 Number of discharged patients	.245	−.072	−.036	.190
X6 Cure rate	.085	−.539	.043	−.005
X7 Improvement rate	−.082	.535	−.035	.017
X8 Clinical and pathological diagnostic accuracy rate	−.059	−.013	.609	.003
X9 Outpatient and hospital admission diagnostic accuracy rate	−.019	−.080	.610	−.001
X10 Bed turnover times	.089	.042	.040	.870
X11Average length of stay	.027	.010	.035	−.406
Extraction method: principal component analysis.				
Rotation method: varimax with Kaiser normalization.				

We establish the factor score function as follows:

F1=0.256X1+0.250X2+0.231X3+0.242X4+0.245X5+0.085X6−0.082X7−0.059X8−0.019X9+0.089X10+0.027X11

F2=−0.045X1−0.025X2−0.022X3−0.083X4−0.072X5−0.539X6+0.535X7−0.013X8−0.080X9+0.042X10+0.010X11

F3=−0.026X1−0.025X2−0.006X3−0.003X4−0.036X5+0.043X6−0.035X7+0.609X8+0.610X9+0.040X10+0.035X11

F4=−0.024X1−0.016X2−0.061X3−0.024X4+0.190X5−0.005X6+0.017X7+0.003X8−0.001X9+0.870X10−0.406X11

The original data of X1 to X11 were standardized values using SPSS (Chicago, IL, USA).

### Calculated score of comprehensive service capabilities

In Table 2 “Total Variance Explained,” the four common factor scores were weighted sums and the weight referred to each factor’s variance contribution rate. The variance contribution rates of the four common factors (F1, F2, F3, and F4) were 33.152%, 17.770%, 12.761%, and 9.758%, respectively. The total variance contribution rate of the four common factors was 73.442%. The comprehensive score function for services capability is as follows:

F=(33.152% F1 + 17.770% F2 + 12.761% F3 + 9.758% F4)/73.442%.

## Results

### Common factor scores in pilot and non-pilot hospitals

The common factor scores are shown in Table 5. F1 represents the hospital scale and service quantity factor. F2 refers to the treatment quality factor. F3 represents the diagnostic quality factor. F4 was called the hospital services efficiency factor. “0” represents the average level of the study samples, a score larger than 0 indicates above average level, and a negative value represents below average level. Because of the characteristics of the factor analysis method, data have been standardized before calculating the factor scores (Table 5).

**Table 5:** Descriptive statistics for common factors (F1, F2, F3, F4) scores between pilot and non-pilot hospitals (*x̄* ± SD)

***Common factors***	***Polit***		***Non-pilot***	
	2009	2012	2009	2012
F1	0.1158±0.9671	0.5300±1.2446	−0.1262±0.9542	0.1136±0.6179
F2	0.0663±0.9846	0.1367±1.0007	−0.0530±1.0373	0.0210±1.0807
F3	0.0470±0.6440	0.0344±0.7468	−0.0432±0.9116	0.0040±0.9579
F4	−0.0690±0.4684	0.0337±0.3981	−0.0180±0.5594	0.0620±0.5738

The comparing results on common factor scores between pilot and non-pilot hospitals are shown in Table 6. For F1, the (mean ±SD) of the different values of factor score between 2009 and 2012 were 0.4142±0.3520 (pilot group) and 0.3468±0.4624 (non-pilot group). The difference between 2009 and 2012 was normally distributed within each group and the two population variances were equal at the significant level 0.05 (F=0.002, *P*=0.964). The t-test of two independent-samples resulted in t=1.579, *P*=0.115.

**Table 6:** The comparing result on common factor scores between pilot and non-pilot hospitals

***Common factors***	***Group***	*d̄*_2012–2009_±*S_d_*	***t***	***P***
F1	Polit	0.4142±0.3520	1.579	0.115
	Non-pilot	0.3468±0.4624		
F2	Polit	0.0704±0.5945	−0.053	0.958
	Non-pilot	0.0739±0.6602		
F3	Polit	−0.0126±0.4905	−1.219	0.224
	Non-pilot	0.0472±0.4523		
F4	Polit	0.1027±0.3043	0.606	0.545
	Non-pilot	0.0800±0.4080		

For F2, the (mean ±SD) s of the different values of factor score between 2009 and 2012 were 0.0704±0.5945 (pilot group) and 0.0739±0.6602 (non-pilot group). The difference between 2009 and 2012 was normally distributed within each group and the two population variances were equal at the significant level 0.05 (F=0.187, *P*=0.666). The t-test of two independent-samples resulted in t=−0.053, *P*=0.958.

For F3, the (mean ±SD) s of the different values of factor score between 2009 and 2012 were −0.0126±0.4905 (pilot group) and 0.0472±0.4523 (non-pilot group). The difference between 2009 and 2012 was normally distributed within each group and the two population variances were equal at the significant level 0.05 (F=0.007, *P*=0.935). The t-test of two independent-samples resulted in t=−1.219, *P*=0.224.

For F4, the (mean ±SD) s of the different values of factor score between 2009 and 2012 were 0.1027±0.3043 (pilot group) and 0.0800±0.4080 (non-pilot group). The difference between 2009 and 2012 was normally distributed within each group and the two population variances were equal at the significant level 0.05 (F=0.372, *P*=0.542). The t-test of two independent-samples resulted in t=0.606, *P*=0.545.

### Comprehensive service capabilities scores in pilot and non-pilot hospitals

The calculation results of the comprehensive factor scores (F) of the service capabilities are shown in Table 7. “0” represents the average level of the study samples, whereas a negative value represents below average level. In pilot and non-pilot hospital,

**Table 7:** Comprehensive service capabilities scores (F) from 2006 to 2012

***Group***	***x̄±SD***	***t***	***P value***
Pilot group			
2009	0.0673±0.5111	−3.662	0.000
2012	0.2828±0.6158		
Non-pilot group			
2009	−0.0800±0.5144	−3.27	0.001
2012	0.1136±0.6179		
2009			
Pilot group	0.0673±0.5111	2.76	0.006
Non-pilot group	−0.0800±0.5144		
2012			
Pilot group	0.2828±0.6158	2.639	0.009
Non-pilot group	0.1136±0.6179		

In pilot hospitals, the F scores were normally distributed both in 2009 and in 2012. The two population variances were not equal at the significant level 0.05 (F=6.217, *P*=0.013). The correction t-test of two independent-samples resulted in t=−3.662, *P*=0.000.

In non-pilot hospitals, the F scores were normally distributed both in 2009 and in 2012. The two population variances were equal at the significant level 0.05 (F=3.423, *P*=0.065). The t-test of two independent-samples resulted in t=−3.270, *P*=0.001.

In 2009, the F scores were normally distributed both in pilot and non-pilot hospitals. The two population variances were equal at the significant level 0.05 (F=1.071, *P*=0.301). The t-test of two independent-samples resulted in t=0.367, *P*=0.006.

In 2012, the F scores were normally distributed both in pilot and non-pilot hospitals. The two population variances were equal at the significant level 0.05 (F=1.350, *P*=0.246). The t-test of two independent-samples resulted in t=2.639, *P*=0.009.

The comparing results on comprehensive service capabilities scores (F) between pilot and non-pilot hospitals are shown in Table 8. For F, the (mean ±SD) s of the different values of factor score between 2009 and 2012 were 0.2151±0.1770 (pilot group) and 0.1930±0.2523 (non-pilot group). The difference between 2009 and 2012 was normally distributed within each group and the two population variances were equal at the significant level 0.05 (F=1.255, *P*=0.263). The t-test of two independent-samples resulted in t=0.976, *P*=0.330.

**Table 8:** The comparing result on comprehensive service capabilities scores (F) between pilot and non-pilot hospitals

	***Group***	*d̄*_2012–2019_±*S_d_*	***t***	***P value***
F	Polit	0.2151±0.1770	0.976	0.330
	Non-pilot	0.1930±0.2523		

## Discussion

The differences from 2009 to 2012 of common factor scores (F1, F2, F3, F4) between pilot and non-pilot hospitals were not statistically significant (P>0.05). There was a difference on the improvement from 2009 to 2012 of F1 (hospital scale and service capacity), F2 (treatment quality), F3 (service quality) and F4 (services efficiency) of sample hospitals between pilot and non-pilot groups. The reform could not have improved fully county public hospitals’ scale and service quantity, treatment quality, service quality and services efficiency.

The comprehensive service capabilities score (F) of hospitals in 2012 was better than that in 2009 either in pilot or non-pilot group (*P*<0.05). Meanwhile, the pilot hospitals’ comprehensive service capabilities score (F) was better than that in non-pilot groups either in 2010 or 2012 (*P*<0.05). The comprehensive service capabilities score (F) of both pilot and non-pilot group all got improvement with the development and progress of socio-economic level, improvement of medical technology and serviceability, and the implementation of the new healthcare reform.

The government increased financial input in the construction of county hospitals, especially the pilot ones. After implementing the new healthcare reform, the coverage of the New Rural Cooperative Medical Scheme, medical insurance for urban workers, and medical insurance for urban residents improved. The medical service demand from the rural population increased and also contributed to the scale expansion of county hospitals. From 2009 to 2012, in both pilot and non-pilot county hospitals, the scale (including number of beds and medical staffs) increased rapidly. The service quantity (including number of outpatient and emergency patients, operations and discharged patients) and the treatment quality (including cure rate and improvement rate) increased. The diagnostic quality (including clinical and pathological diagnostic, outpatient and hospital admission diagnostic) in pilot hospitals decreased, while that in non-pilot hospitals increased. The services efficiency (including bed turnover times, average length of stay) also increased. Judging from the overall trend, the comprehensive service capabilities score (F) of both pilot and non-pilot group all got improvement.

However, the differences from 2009 to 2012 of comprehensive service capabilities score (F) between pilot and non-pilot hospitals were not statistically significant (*P*>0.05). The county public hospital reform did not significantly play a due good role in improving the service capability in pilot group which is one of the reform’s goals. Since China has implemented health care reform in 2009, many studies and papers relating to the reform has published both in Chinese journals and international journals. Most of these papers showed the positive results of the reform. However, this study indicated that the reform has shortcomings. This study evaluated the public hospital reform from a more comprehensive, objective and unique perspective, getting a different point of view.

One of the important reasons was China health reform policy was paying more attention to inputs rather than outputs for pilot hospital and lack of evaluation and supervision on hospital running or operation. Besides, the decision-making department of public health made the decision of how much should be in each hospital by experience management without attaching importance to equity and efficiency, as well as quality (27). Governments should ensure that resources are allocated according to need (4).

Studies in other countries also showed the similar problems. A study analyzing Mexico’s health system (28) concluded that the measures implemented to reform the Mexican health system have failed to achieve the intended results; on the contrary, they have led to a reduction in interventions, rising costs, and a decrease in the installed capacity and professional personnel for the system’s operation. Health systems face new challenges, inevitably requiring that the analyses be situated in a broader framework. In Sweden (29) in terms of structure, process and outcomes mentioned that resource allocation to PHC has become more dependent on provider location, patient choice and demand, and less in need of care. This situation needs to be carefully monitored and countered where necessary. In Ghana, Central government regulations for resource allocation and use should be more flexible, to make services better respond to local needs (30).

These experiences and lessons in this study are meaningful and noteworthy for other counties, especially developing countries. The new suggestions for health care system of other countries are as follows: 1) paying more attention to outputs rather than inputs. 2) Fully evaluation and supervision on health system running, and the equity, efficiency, quality of health system. 3) The decision-making department of public health should make the decision of input according to equity and efficiency, as well as quality, rather than by experience management.

## Conclusion

The comprehensive service capability of both pilot and non-pilot group all got improvement. However, county public hospital reform did not significantly play a due good role in improving the service capability in pilot group. The reform was helpful to improve the hospital current situation, but it has not completely achieved policy objectives in the sample hospitals of this study.

## Ethical considerations

Ethical issues (Including plagiarism, informed consent, misconduct, data fabrication and/or falsification, double publication and/or submission, redundancy, etc.) have been completely observed by the authors.
